# Image-Based Awareness Campaign and Community Mobilization in the Control of Schistosomiasis

**DOI:** 10.3390/tropicalmed8060309

**Published:** 2023-06-05

**Authors:** Louise Makau-Barasa, Liya Assefa, Moses O. Aderogba, David Bell, Jacob Solomon, Abubakar Abba, Juliana A-Enegela, James G. Damen, Samuel Popoola, Jan-Carel Diehl, Gleb Vdovine, Temitope Agbana

**Affiliations:** 1 The Ending Neglected Diseases (END) Fund, New York, NY 10016, USA; lmakau-barasa@endfund.org (L.M.-B.); lassefa@end.org (L.A.); maderogba@end.org (M.O.A.); 2 Independent Consultant, Lake Jackson, TX 77566, USA; bell00david@gmail.com; 3 Federal Ministry of Health, Abuja 900242, Nigeria; owukpa2000@gmail.com; 4 FCT Public Health Department, Abuja 900001, Nigeria; abubakarabbam@gmail.com; 5 CBM International, Cambridge CB5 8HY, UK; juliana.amanyi-enegela@cbm.org; 6 Medical Lab Department, University of Jos, Jos 930001, Nigeria; jamesgdamen1@gmail.com; 7 AiDx Medical Bv, 2641 KM Pijnacker, The Netherlands; greatsampop@gmail.com (S.P.); gleb.vdovin@gmail.com (G.V.); 8 Sustainable Design Engineering, Delft University of Technology, 2628 CE Delft, The Netherlands; J.C.Diehl@tudelft.nl

**Keywords:** schistosomiasis, community mobilization, awareness, public health, treatment, control, elimination

## Abstract

Community awareness and participation in mass screening is critical for schistosomiasis control. This study assessed the impact of sharing anonymized image-based positive test results on the uptake of screening during community mobilization outreach. We conducted an observational study to compare the population response to standard and image-based strategies in 14 communities in Abuja, Nigeria. Six hundred and ninety-one (341 females, 350 males) individuals participated in this study. We analyzed the response ratio, relative increase, and sample collection time. The potential treatment uptake and change in social behavior were determined based on a semi-structured questionnaire. The mean response ratio of the image-based strategy was 89.7% representing a significantly higher ratio than the 27.8%, which was observed under the standard mobilization approach (*p* ≤ 0.001). The image-based method was associated with 100% of the participants agreeing to provide urine samples, 94% willing to be treated, 89% claiming to have been invited to participate in the study by a friend, and 91% desiring to change a predisposing behavioral habit. These findings indicate that image-based community awareness campaigns may increase the population’s perception about schistosomiasis transmission and treatment. This raises new possibilities for local resource mobilization to expand services in reaching the last mile in schistosomiasis control.

## 1. Introduction

Schistosomiasis is a snail-borne acute and chronic parasitic disease that is caused by trematode blood flukes of the genus *Schistosoma* [1,2]. Globally, it is one of the World Health Organization’s (WHO) neglected tropical diseases (NTDs) that has increasingly drawn the attention of public health experts over the past decade and a half. Transmission has been reported from 78 countries, the majority of which are classified as low- or middle-income countries, according to a WHO report [3]. There are two major forms of schistosomiasis—intestinal and urogenital. Most human infections in Africa are caused by *Schistosoma mansoni* (intestinal) or *S. haematobium*.

Common signs and symptoms of *S. haematobium* include a swollen belly, blood in the urine, stunted growth, cognitive impairment in children, and infertility among adults of childbearing age [4,5]. Advanced disease may sometimes present with fibrosis of the bladder and ureter, kidney damage, and bladder cancer [6,7].

Several sociocultural risks and environmental factors are found to influence schistosomiasis infection and prevention [8,9]. Limited knowledge of transmission modes, signs, symptoms, diagnosis, and prevention also affects efforts to control schistosomiasis [4,10]. A low level of knowledge and poor adherence to recommended practices tend to influence risky practices, such as defecation and urination in water, swimming, bathing, and fishing [11,12]. Due to the low level of awareness, community leaders and members are reported to show a negative response towards control intervention programs, and fail to undertake social behaviors that could mitigate disease transmission, according to Sacolo et al. [4,13,14].

Mass drug administration (MDA) with praziquantel is one of the approved strategies for the control of schistosomiasis in at-risk populations. The uptake of treatment, however, suffers from low awareness of the consequences of the disease, low morbidity, inadequate health education, and uncomfortable side effects [15,16,17,18,19,20].

Health communication campaigns use mass media and an organized set of activities to generate specific outcomes or influence a large number of individuals. Such awareness campaigns include community announcements, which are delivered through a different combination of communication channels, including road shows, posters, information, and education and communication (IEC) materials (small booklets and brochures) containing information about schistosomiasis.

Various health communication campaigns have been used to affect human behaviors in areas such as reproductive health, human immunodeficiency virus (HIV)/acquired immunodeficiency syndrome (AIDS), malaria, and sex-related behaviors. However, very little experience with these interventions has been documented for the mass administration of medicines for the control of schistosomiasis [21].

The standard awareness and community mobilization method currently used for sensitization of school-aged children and adults in Abuja, Nigeria is the IEC. These information education and communication materials typically contain pictures of worms, kids swimming in the water, urine, and some textual information about schistosomiasis, prevention, signs and symptoms.

The impact of an awareness and community mobilization approach that includes the direct demonstration of live test results from the target population on key components of control and intervention programs has not been investigated. To address this gap, we designed a data-driven experiment and a survey questionnaire that included the presentation of digital images of analyzed urine samples of patients in the community. 

The primary objective of this study was to assess the potential increase in awareness and community participation in the testing and treatment of *S. haematobium* when anonymized actual image-based test results from endemic communities were shared with the affected population during community mobilization outreach.

## 2. Materials and Methods

### 2.1. Ethics Statement

Ethical approval was received from the Federal Capital Territory (FCT, Abuja, Nigeria) Health Research Ethics Committee (FCT, HREC) under the following approval number: FHREC/2022/01/102/05-07-22. The project was introduced in detail to the NTD Unit, Public Health Department, Federal Capital Territory Abuja (FCTA). 

The questions asked in the survey aimed at determining the awareness level of schistosomiasis among school-aged children. The mode of transmission and specific health implications associated with the disease were assessed through the questions in the survey. The survey also focused on understanding the perspectives of the participants when exposed to the images of the schistosome ova and the consequent impact on their willingness to change a behavioral habit that could lower the risk of infection.

Following standard government protocol, the research goals and objectives were explained to each community leader, local authorities, and residents. Written informed consent was obtained from the community leaders in each community in lieu of individual consent and was approved by the FCTA NTD public health data manager. 

The participant confidentiality was ensured by allocating patient ID to each of the patient samples. The collected samples were labeled with a sample identification number. Furthermore, the interview questions were administered by the Area Council NTD staff located in the community under the supervision of the state FCTA NTD public health data manager. In total, 4 community leaders and 65 school-aged children (SAC) with an average age of 10 years participated in the interview.

### 2.2. Sample Size

Using the Cochran formula ($N_{O\;}$= $\frac{Z^{2}pq}{e^{2}}$), we computed the sample size of the study population, where e is the desired level of precision at the 95% confidence interval (i.e., the margin of error), p is the baseline prevalence (estimated proportion of the population, which has the attribute in question), and q is 1 − p. The Z values at 95% confidence level provided us a value of 1.96. Given the baseline prevalence for Gwagwalada and AMAC at 51.9% and 21.12%, respectively, a total of 691 (350 males and 341 females) respondents were randomly sampled from 9 and 5 communities in Gwagwalada and AMAC, respectively.

### 2.3. Study Area and Participant Selection

**Figure 1:**
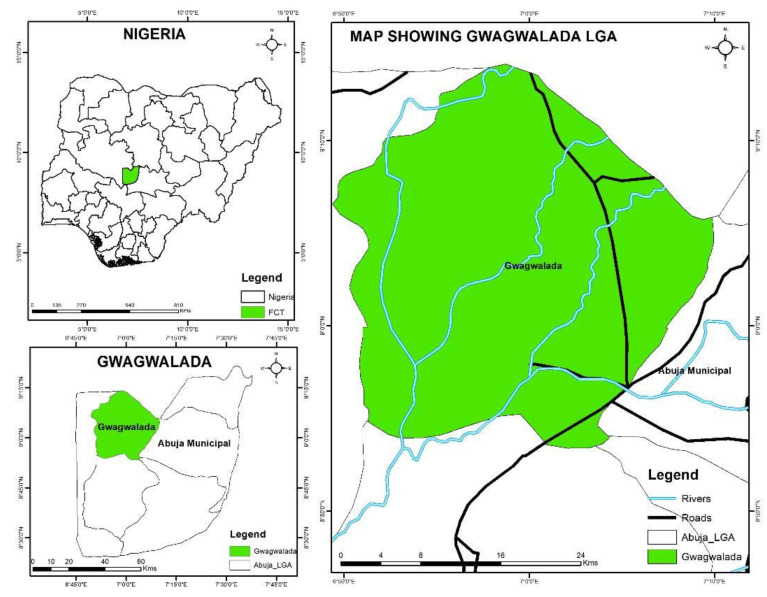
Map of the six Abuja area councils, showing the selected study area of Gwagwalada and AMAC.

Participants aged 5 and above were eligible to participate in this study. The study was conducted in 9 communities in the Gwagwalada Area Council and 5 communities in the Abuja Municipal Area Council (AMAC) in Abuja, FCT Nigeria. In total, 7 of the 9 communities in Gwagwalada are located in rural areas and 2 are located in urban areas. The Abuja Municipal Area Council (AMAC) is one of the six area councils in the Federal Capital Territory Abuja, Nigeria, as shown in Figure 1. Study participants were drawn from 5 rural communities in the AMAC area council. The main economic activity of the community members is subsistence agriculture. Additionally, the approximate population size, location, and coordinates of all selected communities are included in Table 1. A total number of 691 school-aged children from the communities participated in this study.

The area councils were specifically selected based on the disease prevalence information extracted from the baseline database of the Federal Ministry of Health NTD department on schistosomiasis. The participants collected their urine samples in the bathroom at collection sites. These were stored in temperature-controlled storage boxes at 18 °C and transported to the laboratory immediately after collection.

### 2.4. Study Design

The study was divided into two main phases. The first phase of the research was a purposive selection of 50 pupils with a current history of hematuria from two communities known to be endemic for schistosomiasis based on available mapping data. The second phase was the observational study conducted in 14 other communities within the same area councils using the standard mobilization approach (SMA) and the proposed image-based intervention. The community consent forms were filled and signed by community leaders on behalf of members of their communities according to standard government protocol. The consent forms were administered by the data manager of the NTD unit, the Public Health Department of the Federal Capital Territory Abuja (FCTA). The call for the participants (school-aged children) to participate in the study was made by the head teacher of the school together with the NTD ward, who was the focal person responsible for the schools in the community.

The aim of the first phase of the study was to conduct a microscopy analysis on samples that showed visible hematuria and obtain digital images that would be used for this study in the second phase of the research. In this phase, trained public health and data management experts from the NTD Unit, Public Health Department, FCTA, conducted a pre-sample collection awareness campaign and mobilization in two schools. Digital microscopy analysis on prepared samples was performed using the AiDx NTDx digital microscope. The acquired digital images were printed and used during this study. 

In the second part of this study, the sample collection was community- and school-based. Therefore, the exclusion criteria were limited to unwilling respondents. The same team of experts from the NTD Unit visited each of the selected communities to collect urine samples. Two advocacy and community mobilization strategies were administered to the same audience to motivate sample collection at the collection site. The standard mobilization approach was first administered using the standard information education and communication materials. The number of participants willing to provide their urine samples for analysis was recorded. Thereafter, the image-based approach (IMA) (SMA with a digital image of actual test results from neighboring communities as supporting evidence) was presented to the same audience. 

**Figure 2:**
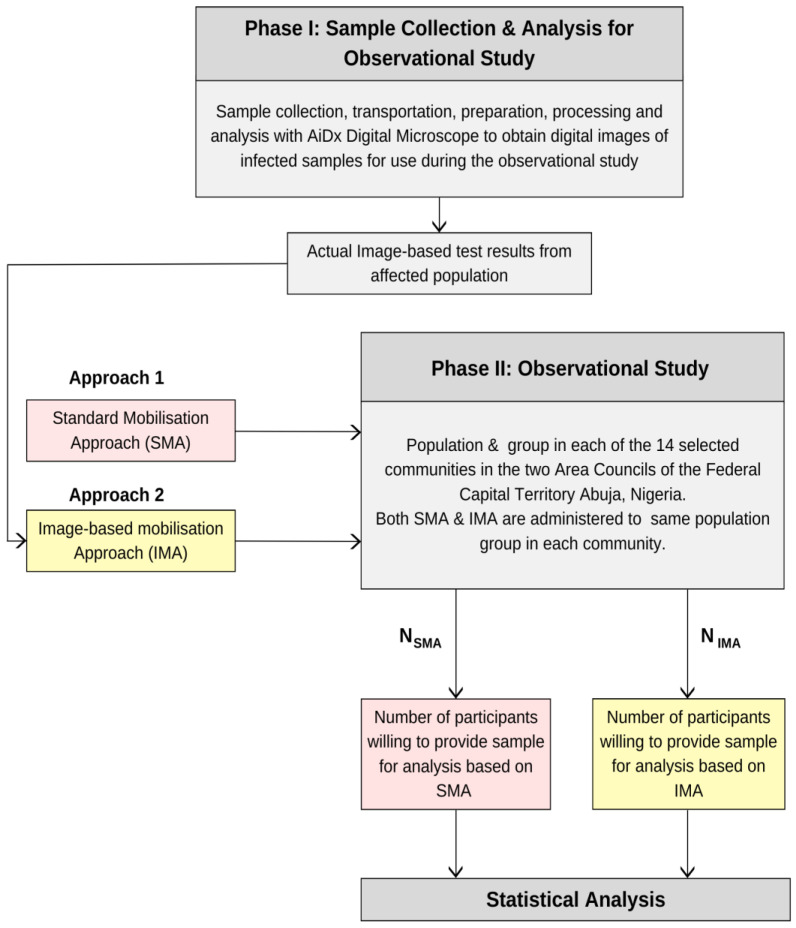
Description of sample collection in phase I & II and the process for the observational study.

A second call for participants to provide urine samples for analysis was made using IMA, and the number of willing participants from the same audience was recorded. In some communities, interviews were conducted during advocacy and community mobilization, and responses of the community leaders were recorded accordingly. Description of sample collection process in both parts of the study is shown in Figure 2.

The study targeted collecting 55 urine samples per community for a total of 770 urine samples from the 14 communities. To measure the impact on potential treatment uptake and change in social behavior, a semi-structured survey was conducted among the school-aged children in the Gwagwa community of the Abuja Municipal Area Council (AMAC). The questionnaire was administered by the local NTD staff located in the community under the supervision of the state NTD data manager. Overall, 65 school-aged children (SAC) with an average age of 10 years participated in this survey. 

### 2.5. Procedure

Participants were taken through a short demonstration and training (of about one hour) on the use of the AiDx NTDx Assist device using the instruction manual (Tool one). After training, participants were asked to use the device based on what they learned from memory (i.e., without the aid of the user manual) under the observation of a single investigator (Tool two: observational checklist). The observational checklist captures four steps taken in using the device: (a) turning on the machine, (b) starting the application, (c) putting in slides, and (d) reading output results. The device scanning time was programmed for 8 min and not captured by the checklist since it is constant across all participants. Thereafter, participants were asked to fill out the questionnaires (Tools three and four) to document their experience.

The goal of this experiment was to compare the response of participants in providing their samples and to provide an estimate of the level of awareness that is created when community awareness and mobilization are performed based on direct demonstration of anonymized life test results to the target population. The response rate was compared to the standard mobilization approach in this study.

#### 2.5.1. Standard Mobilization Approach

The standard mobilization approach (SMA) is based on a verbal introduction of schistosomiasis, its mode of transmission, its symptoms, and consequences if left untreated. The end-result of the SMA is to sensitize the respondents, motivate community awareness, increase treatment and uptake of medicine, and stimulate their willingness to provide their urine or stool samples for diagnostic analysis. The results are typically used to create treatment and logistics plans for MDA and community education within the Water, Sanitation and Hygiene (WASH) program. As part of the SMA, labeled urine collection sample bottles are immediately distributed to interested participants for diagnostic analysis. 

In this study, 55 labelled urine collection bottles were prepared for each community. The estimated number of willing participants that offered to provide their urine samples after the SMA presentation was documented. 

#### 2.5.2. Image-Based Approach

**Figure 3:**
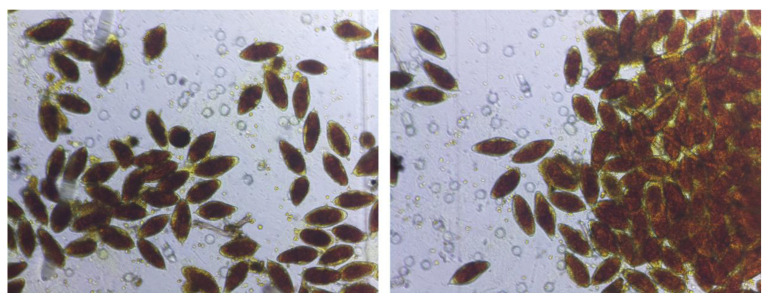
AiDx NTDx results of registered digital images of filtered urine based on a sample of urine from one of the study participants, a school-aged child in the Gwagwalada Area Council. This image and a few others were used for image-based advocacy and community mobilization in addition to the standard information, education, and communication material.

The second approach (the image-based mobilization approach (IMA)) involved the presentation of the standard mobilization approach to the same audience that participated in the SMA using registered de-identified images, such as those shown in Figure 3, as supporting evidence. The mobilization approach was made with an emphasis on the fact that the demonstrated images were actual test results of a sample collected from the school children in a nearby community. After the presentation, a second call for participants to willingly provide their samples for disease diagnosis and potential follow-up treatment was made. The response to the second call was estimated by counting the number of willing participants who collected the urine sample bottle. The estimated value was immediately documented by the recorder. The process was repeated in the remaining 13 communities. 

To compute the relative increase with respect to the SMA value, we divided the absolute difference between the SMA and IMA values by the SMA value ($\frac{\left(N-O\right)}{O})$, where O is the SMA value and N is the IMA value recorded per community. The response ratio of both the SMA and IMA for each community was computed by dividing the number of actual responses by the number of expected responses. Based on our sampling technique, the expected response number for each community was set at 55.

### 2.6. Data Analysis

Descriptive statistics were conducted to compare the SMA and the IMA. T-test analyses were conducted to examine if there were any statistical significance in the response ratio of the SMA and IMA techniques. The data analysis was performed using MATLAB (© 1994–2023 The MathWorks, Inc., Natick, MA, USA) and DATAtab: Online Statistics Calculator, Seiersberg, Austria.

## 3. Results

### 3.1. Demographics

A summary of the demographic characteristics of the study population is provided in Table 2. A total of 691 participants stratified by school-aged children (5–12 years old), adolescent/young adults (13–18 years old), and adults (19 years old) were selected from the 14 communities in the Gwagwalada and AMAC area councils of the Federal Capital Territory, Abuja, Nigeria. Overall, the age of the participants ranged from 5–60 years. School-aged children between the ages of 5–12 years constituted 81.3% of the total sample population. The difference in male to female participants was not significant (350 males versus 341 females).

Table 2 depicts the number of voluntary participants recorded in the selected communities after using the SMA and IMA, respectively. The computed response ratios based on the SMA and the IMA values are presented in Table 3. The image-based community mobilization achieved significantly higher response rates than the standard method (mean of 89.74% for IMA [72, 100] vs. 27.85% for SMA [7.2, 49], *p* ≤ 0.001).

### 3.2. Response Ratio

The IMA model demonstrated a significantly high response ratio of 100% and a minimum response ratio of 72.7%, as shown in Table 4. The highest response ratio for the standard advocacy method was 49% in the Dagiri community. Furthermore, a notable difference in both methods was observed in the Ruga Fulani community, where the SMA had a response ratio of 7.2% as compared to a 90.9% response ratio in the same community after the use of image-based mobilization. The difference between uptake through the standard and image-based approaches was statistically significant (Levene’s test, *p* < 0.001).

### 3.3. Sample Collection Time

The standard advocacy approach requires an average time frame of 210 min of community mobilization for the collection of approximately 50 urine samples in the selected communities, as reported by the data manager from the NTD Unit. With the image-based method, however, more than 50 urine samples were collected in less than 90 min. Thus, we observe an increase in time efficiency of the public health field workers. Additionally, a potential increase in the uptake of treatment was observed, as more than 70% of the community members asked questions about treatment possibilities and timing for treatment.

### 3.4. Community Acceptance

The community leaders of eight out of the selected fourteen communities visited initially showed some reluctance and passivity in mobilizing their community members to participate and provide their urine samples for laboratory analysis in this study. The research team, using the proposed IMA, observed a positive response and interest in the community leaders as they became more interested in understanding the mode of transmission of the disease. One high-level community leader who instantly mobilized his aides to take note of the discussion and prepare the community members to participate in the study stated as follows:

*“I have never been confronted with the reality of the impact of this disease in my community like this. No one has ever shown me an image or a picture like this. This picture shows that our kids are in danger and need to now find a way to stop them from playing and interacting with the infected water bodies.”*

### 3.5. Potential Treatment Uptake and Change in Social Behavior

Of the 65 children that answered the questionnaire, 24 (37%) were male and 41 (63%) were female. In total, 80% of the respondents reported having heard about schistosomiasis and its specific health implications, while 15% knew nothing about the disease. Moreover, 71% of the students knew that disease transmission could occur through contact with rivers and other water bodies in their community. Additionally, 47 out of the 65 respondents confirmed that they see blood in their urine and 43% of this population informed their parents about their condition. However, only 3% of the respondents were taken to the hospital for medical attention.

When asked about how they feel after seeing the images of the schistosome ova of other infected children in their community (patient data were treated with uttermost privacy and confidentiality), 89% had negative emotional responses. Words such as “fear, sadness, death, scared, feeling of pain” were common in their responses. As a result, 100% of the respondents were willing to provide urine for medical testing and analysis to determine their health status and 94% expressed a desire to have themselves and their family members treated urgently. When questioned about what social behavioral change might prevent the spread of the disease, 91% said they will not go to the river to swim or bathe anymore. To measure the impact of community awareness, we asked how they were informed about the sample collection exercises. Based on this, 89% claimed to have been invited and one respondent claimed to have invited eight of his friends. 

### 3.6. Local Resource Mobilization

Based on the IMA, individuals were concerned by the images and committed personal resources toward the treatment of school-aged children in the communities discussed. A commitment to provide feeding and deworming to 1160 children in the communities has been received, and more individuals are willing to donate after viewing the images.

## 4. Discussion

To the best of our knowledge, this is the first observational study to observe the response rate of IMA methods in an awareness campaign that could motivate treatment uptake, change in social behavior, and local resource mobilization. 

With the current standard community awareness and mobilization approach, motivating behavioral changes among school-aged children has been a very challenging task. Interaction of school-aged children with water bodies through swimming, playing, and bathing has been a major problem for the control and elimination interventions of the schistosomiasis program. Cases of reinfection after years of mass administration of medicines (MAM) are widely reported. From the result obtained from this study, however, our image-based mobilization approach has shown some promising potential to create awareness and community participation in the control and elimination efforts. 

Ninety-one percent (91%) of the school-aged children who participated promised not to swim in the river after being confronted with the live test results that vividly displayed the schistosome ova. This is a preliminary result of the intentions of the participants. We aim to validate the impact of this outcome on real-time social behavior with data from a larger study. In addition, local resource mobilization has been a major challenge in the control and elimination of schistosomiasis in endemic countries. 

Through this research work, we have demonstrated that showing people live test results could potentially raise resources that would contribute positively to schistosomiasis and soil-transmitted helminths control and elimination programs. 

Frequent exposure and access to clear, consistent messages from credible channels and sources will raise household awareness about schistosomiasis, but live test results from known communities have been shown here to further improve awareness. From this study, we infer that the image-based awareness and mobilization approach could potentially increase uptake of the treatment and awareness and motivate change in social behavior among school-aged children. Community leaders in a majority of communities who had initially shown reluctance and passivity in mobilizing their community members to participate and provide their urine samples demonstrated an increased interest and understanding of disease transmission, which is something that was not achieved through previous standard mobilization approaches and familiarity with local NTD coordinators. 

Adults and children in all communities showed a marked increase in participation when they were able to view actual images of disease evidence in urine from nearby communities. It is likely that both the visual stimulus (obvious foreign objects that have come from the body) and the close geographical proximity are seen as having greater relevance than general, relatively depersonalized messaging that they are aware is broadcasted to much of the country. Proof of local transmission would seem to produce more urgency than a standardized national campaign that listeners know is also tailored for very different communities.

The relative ease of obtaining images with mobile devices, such as the AiDx device used here, offers the opportunity to scale this approach. Images could be taken at the start of a regional campaign or in initial sampling in a specific community, and thus provide both geographic and temporal relevance to the target population. The research team here has developed the capability to undertake similar imaging for *S. mansoni* in fecal matter, and the current approach will also be suitable for *S. japonicum and S. mekongi.*

## 5. Strengths and Limitations

Administering the SMA before the IMA intervention could have influenced the response rate of the IMA and introduced a bias to the overall results. Parallel administration of both approaches could probably produce a different result. However, the increase in the interest recorded from community leaders, the qualitative feedback from interviews, and the result obtained by the FCTA, Department of Public Health, NTD unit, indicate that the overall impact of the IMA is real. The study was strengthened by the large sample of participants and was relatively balanced between genders. 

The number of school-aged children that participated in the survey was low. A larger study is required in this age group to confirm the impact of images on childhood recruitment; in this larger study, a cross-observational study method would be appropriate.

## 6. Conclusions

In this paper, we have demonstrated that the direct demonstration of anonymized actual test results (digital images of *Schistosoma haematobium*) to the affected group/population during community mobilization and awareness campaigns could improve community response and corresponding uptake of diagnosis and treatment. In a coordinated approach, these images can contribute to better planning by communities and state health authorities, minimize the wastage of material resources (e.g., sample collection bottles), and potentially motivate social behavior change in at-risk communities. We infer that the proposed approach could bridge the knowledge gap and motivate change in attitude and practice. 

Based on the results obtained from this study, the NTD Unit, Public Health Department, FCTA, has adopted the IMA approach for community mobilization, schistosomiasis education and awareness campaigns, and advocacy in their October 2022 mass administration of medicine in the endemic areas of the Federal Capital Territory, Abuja.

## Figures and Tables

**Table 1:** Communities, approximate population size, and coordinates of the locations where the samples were collected.

Community	Area Council	Population	Latitude	Longitude
Kpaipai	AMAC	3860	8.491722	7.291574
Ibwa	Gwagwalada	2050	9.0637061	7.0585739
Kpakuru Serki	Gwagwalada	410	9.007916	7.201145
Ruga Fulani	AMAC	4850	8.960753	7.334015
Dagiri	Gwagwalada	5800	8.932408	7.085754
Angwa Dodo	Gwagwalada	7200	8.939232	7.095248
Bassan Jiwa	AMAC	6700	9.018901	7.285971
Kpakuru	Gwagwalada	620	9.01886	7.191984
Angwa Bassa	Gwagwalada	4600	8.935981	7.084876
Gwagwa	AMAC	8500	9.088100	7.317062
Karmo	AMAC	4500	9.069595	7.363901
Dobi	Gwagwalada	3450	9.056181	6.991864
Paiko	Gwagwalada	2200	8.996822	7.027274
Dukpa	Gwagwalada	1300	8.989871	7.078443

**Table 2:** Characteristics of the study population in Gwagwalada and AMAC area council of the FCTA.

Age Group	Male (%)	Female (%)	Total (%)
5–12	303 (43.85)	259 (37.48)	562 (81.33)
13–18	47 (6.80)	22 (3.18)	69 (9.99)
19+	0 (0.00)	60 (8.68)	60 (8.68)
	350 (50.65)	341 (49.35)	691 (100)

**Table 3:** Community response to standard mobilization approach and image-based mobilization approach.

Community	Standard Mobilization Approach (SMA)	Response Ratio Based on SMA (%)	Image-Based Mobilization Approach (IMA)	Response Ratio Based on IMA (%)	Relative Increase (IMA − SMA)/ SMA (%)
Kpaipai	8	14.5	55	100	587.5
Ibwa	16	29	40	72.7	150
Kpakuru Serki	10	18.1	42	76.4	320
Ruga Fulani	4	7.2	50	90.9	1150
Dagiri	27	49	53	96.4	96.3
Angwa Dodo	21	38.2	50	90.9	138.1
Bassan Jiwa	24	43.6	50	90.9	108.3
Kpakuru	13	23.6	51	92.7	292
Angwa Bassa	12	21.8	48	87.2	300
Gwagwa	25	45	50	90.9	100
Karmo	7	12.7	49	89.1	600
Dobi	17	30.9	50	90.9	194.1
Paiko	10	18.18	53	96.4	430
Dukpa	21	38.18	50	90.9	138.1

**Table 4:** Mean participation achieved through standard and image-based approaches across the study communities.

	Standard Mobilization Approach (SMA, %)	Image Mobilization Approach (IMA, %)	Response Ratio Based on SMA (%)	Response Ratio Based on IMA (%)
Mean	15.36	49.36	27.85	89.74
Std. Deviation	7.32	3.99	13.28	7.26
Minimum	4	40	7.2	72.7
Maximum	27	55	49	100

## Data Availability

The datasets generated and analyzed during the current study are not publicly available but are available from the corresponding author upon reasonable request.
